# Transforming cancer clinical trials: The integral role of artificial intelligence in electronic health records for efficient patient recruitment^☆^

**DOI:** 10.1016/j.conctc.2023.101223

**Published:** 2023-11-07

**Authors:** Abdulqadir J. Nashwan, Salam Bani Hani

**Affiliations:** a Director of Nursing for Education & Practice Development, Hamad Medical Corporation, Doha, Qatar; b Department of Public Health, College of Health Sciences, QU Health, Qatar University, Doha, Qatar; c Faculty of Nursing, Nursing Deparment, Irbid National University, Irbid, Jordan

**Keywords:** Artificial intelligence, Electronic health records, Cancer, Clinical trials, Subject recruitment

## Abstract

Healthcare is one of the sectors where artificial intelligence (AI) is currently viewed as a crucial driving factor. Patient care, medical research, and clinical trial enrollment could all significantly improve due to AI's incorporation into electronic health records (EHRs). This short communication highlights how AI may improve the recruitment process regarding speed, accuracy, and overall cancer clinical trial efficiency. AI can automate this procedure by utilizing machine learning (ML) algorithms, identifying potential trial participants quickly and precisely. Many challenges could be addressed due to this integration, including data privacy and security that can be resolved through cutting-edge encryption techniques and differential privacy algorithms that ensure data anonymization. Another significant obstacle is the lack of common EHR formats and interoperability that can be addressed by creating a standardized structured layout. Automating and improving recruitment processes with AI may speed up research, increase the effectiveness of clinical trials, and open the door to more specialized cancer treatments.

## 1 Revolutionizing cancer clinical trials: the integral role of AI in EHRs for efficient patient recruitment

Healthcare is one of the sectors where artificial intelligence (AI) is currently viewed as a crucial driving factor. Patient care, medical research, and clinical trial enrollment could all significantly improve due to AI's incorporation into electronic health records (EHRs) [1]. This is especially noteworthy in complicated diseases like cancer, where the urgent need for novel and expedited research approaches lies in effectively framing, justifying, and communicating the significance of efficient patient recruitment [2]. This article will explore AI's role in cancer clinical trial patient recruitment through integration with EHRs, looking at how it can improve the recruitment process in terms of speed, accuracy, and overall clinical study efficiency.

EHRs are large databases containing essential patient data, including personal demographic information, medical histories, and past and present treatments [3]. AI can process these massive data sets to yield valuable insights that can be used to improve healthcare delivery and develop new research techniques [4]. However, manual searching through these complex datasets is time-consuming and incredibly ineffective [5]. The value of AI rests in its capacity to evaluate data quickly and precisely, revolutionizing the entire patient recruiting process for clinical trials [6].

A significant impact of AI integration into EHRs for cancer clinical trials is the increased speed and efficiency in patient recruitment. To assess a patient's eligibility for clinical trials, healthcare professionals traditionally methodically review each patient's medical history. However, this traditional way is labor-intensive and time-consuming [7]. AI, using Machine Learning (ML) algorithms, can automate this process, identifying prospective trial participants swiftly and accurately [8]. The algorithms can process complex relationships among numerous health indicators, identifying suitable candidates based on specified criteria. This dramatic increase in interpreting the collected data from the initial idea or conception to the execution of clinical trials will imply an efficient and faster research process. This suggests that the research process will become more flexible and adaptable, which may speed up discoveries and improvements in the treatment of cancer [9,10].

A higher degree of accuracy when choosing patients for clinical trials can be guaranteed by AI [6]. By looking at various health factors, machine learning algorithms can identify which patients benefit most from a particular clinical trial. Due to the potential impact of the applicant's appropriateness on the trial's outcomes, clinical research for cancer must be highly specialized [11]. Personalized medicine, which matches distinct patient attributes with trial eligibility requirements and boosts treatment efficiency and patient outcomes, is another key field that AI enables. Furthermore, AI has the potential to widen the scope of patient recruitment. Geographic and logistic constraints confine traditional recruitment methodologies. AI systems can analyze EHRs and identify eligible patients from an extensive pool, breaking the barriers of geographical locations and varied healthcare providers. AI systems can help in remote patient identification, eliminating the necessity for a patient's physical presence at a particular site. AI can even predict patient adherence and dropout rates, enabling more efficient planning and execution of the clinical trial [12].

Natural Language Processing (NLP), a branch of AI, is crucial in handling the unstructured data included in electronic health records [13]. Symptoms, diagnoses, and treatment outcomes are just a few examples of the essential information that medical notes in the EHR frequently contain in an unstructured format. To better identify patients for cancer clinical trials, healthcare practitioners can extract specific and contextually pertinent data from the patient's EHR using NLP, which can understand this unstructured data [14].

Bias and fairness are important issues as well. Data scientists, ethicists, subject matter experts, and policymakers collaborate on an ongoing, multidisciplinary effort to address prejudice and ensure justice in healthcare ML applications. Prioritizing these issues is crucial to preventing the continuation of current biases and ensuring that healthcare AI technologies serve every person equally [17]. Interoperability and lack of standardized EHR formats also pose significant challenges. Different EHR systems may use different layouts, causing difficulties in exchanging information seamlessly. However, adopting standardization protocols, data exchange frameworks, and AI advancements can address these issues. For instance, the Fast Healthcare Interoperability Resources (FHIR) standardization protocol is used for electronically transmitting medical information by outlining a collection of guidelines, formats, and informational components that make exchanging EHR data easier [18].

**Fig. 1:**
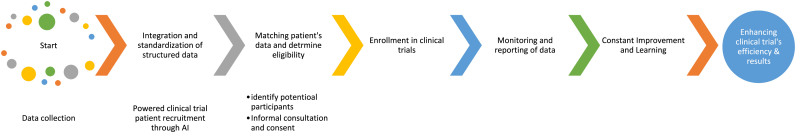
Flow diagram demonstrates the integral role of AI in EHRs for efficient patient recruitment in cancer clinical trials.

To sum up, the recruiting process for cancer clinical trials has the potential to be revolutionized by the integration of AI into EHRs. Automating and improving recruitment processes with AI may speed up research, increase the effectiveness of clinical trials, and open the door to more specialized cancer treatments. These benefits demonstrate the great potential of AI in healthcare, especially in the study and treatment of cancer. It also emphasizes the necessity for ethical and responsible deployment and the significance of addressing issues with data privacy, fairness, and openness. These factors must be considered to guarantee that AI benefits patients and advances healthcare. By pursuing this goal, researchers want to revolutionize cancer research and therapy, opening the door to a time when the disease may be more effectively monitored, treated, and even cured. As the field continues to evolve, the integration of AI and EHRs is certain to play a pivotal role in shaping the future of cancer clinical trials (Fig. 1).

## Ethics approval and consent to participate

Not applicable.

## Funding

None to disclose.

### Authors' contributions

AJN & SBH: Conceptualization, writing-original draft, editing, review-final draft.

All authors read and approved the final manuscript.

## Declaration of generative AI and AI-assisted technologies in the writing process

While preparing this work, the authors used GPT-4 to improve the quality of writing. After using this tool, the author reviewed and edited the content as needed and took full responsibility for the publication's content.

## Declaration of competing interest

The authors declare that they have no known competing financial interests or personal relationships that could have appeared to influence the work reported in this paper.
